# Antibacterial Activity of Some Flavonoids and Organic Acids Widely Distributed in Plants

**DOI:** 10.3390/jcm9010109

**Published:** 2019-12-31

**Authors:** Artur Adamczak, Marcin Ożarowski, Tomasz M. Karpiński

**Affiliations:** 1 Department of Botany, Breeding and Agricultural Technology of Medicinal Plants, Institute of Natural Fibres and Medicinal Plants, Kolejowa 2, 62-064 Plewiska, Poland; artur.adamczak@iwnirz.pl; 2 Department of Biotechnology, Institute of Natural Fibres and Medicinal Plants, Wojska Polskiego 71b, 60-630 Poznań, Poland; marcin.ozarowski@iwnirz.pl; 3 Department of Medical Microbiology, Poznań University of Medical Sciences, Wieniawskiego 3, 61-712 Poznań, Poland

**Keywords:** kaempferol, naringin, orientin, rutin, vitexin, chlorogenic acid, citric acid, malic acid, quinic acid, rosmarinic acid

## Abstract

Among natural substances widespread in fruits, vegetables, spices, and medicinal plants, flavonoids and organic acids belong to the promising groups of bioactive compounds with strong antioxidant and anti-inflammatory properties. The aim of the present work was to evaluate the antibacterial activity of 13 common flavonoids (flavones, flavonols, flavanones) and 6 organic acids (aliphatic and aromatic acids). The minimal inhibitory concentrations (MICs) of selected plant substances were determined by the micro-dilution method using clinical strains of four species of pathogenic bacteria. All tested compounds showed antimicrobial properties, but their biological activity was moderate or relatively low. Bacterial growth was most strongly inhibited by salicylic acid (MIC = 250–500 μg/mL). These compounds were generally more active against Gram-negative bacteria: *Escherichia coli* and *Pseudomonas aeruginosa* than Gram-positive ones: *Enterococcus faecalis* and *Staphylococcus aureus*. An analysis of the antibacterial effect of flavone, chrysin, apigenin, and luteolin showed that the presence of hydroxyl groups in the phenyl rings A and B usually did not influence on the level of their activity. A significant increase in the activity of the hydroxy derivatives of flavone was observed only for *S. aureus*. Similarly, the presence and position of the sugar group in the flavone glycosides generally had no effect on the MIC values.

## 1. Introduction

Screening biological studies of chemical compounds of natural origin allow for assessment of their activity and determine further research stages in order to search for new therapeutic solutions based on active compounds known in plants. This is especially important during the observed increasing resistance of bacteria and fungi to antibiotics. Multidrug resistance (MDR) is a serious threat to human health, but also to crops and animals. MDR is a growing challenge in medicine. Recently, several multinational studies have been carried out to determine the prevalence of herbal medicine use in infections due to pathogenic microorganisms [1,2]. It is considered that extracts of medicinal plants can be an alternative source of resistance modifying substances [2]. It is well known that plant extracts and other herbal products are complex mixtures containing the wide variety of primary and secondary metabolites, and their action may be the result of the synergy of different chemical components. Moreover, these extracts may show various mechanisms of biological and pharmacological activity, i.e., ability to bind to protein domains, modulation of the immune response, mitosis, apoptosis, and signal transduction [2]. However, it should be noted that plants interact with the environment and other organisms, therefore their chemical composition and the level of active substances can be very diverse [3,4]. In addition, the manufacturing process of herbal medicinal products is very complex because it encompasses non-standardized processes like the cultivation of plants, obtaining the vegetable raw material from various parts of the world, preparing of extract, and producing a product in accordance with local guidelines of the good manufacturing practice. Therefore, it can be concluded that using pure chemical compounds of natural origin would be an interesting complementary option due to their easier therapeutic dosage, the study of mechanisms of the pharmacological action and monitoring of their side effects.

A lot of widespread plant substances, including alkaloids, organosulfur compounds, phenolic acids, flavonoids, carotenoids, coumarins, terpenes, tannins, and some primary metabolites (amino acids, peptides, organic acids) exhibit antimicrobial properties [1,5,6,7,8]. Among them, flavonoids are a promising group of bioactive substances with low systemic toxicity. Natural flavonols, flavones, flavanones, and other compounds of this class belong to the common secondary metabolites found in various fruits, vegetables, and medicinal plants [9] showing strong antioxidant and anti-inflammatory properties [10,11]. Dietary polyphenols such as flavonoids and phenolic acids, consumed in large quantities in foods of plant origin, exhibit a number of beneficial effects and play an important role in the prevention of chronic and degenerative diseases. Not only their antioxidant and anti-inflammatory activities, but also neuroprotective, anticancer, immunomodulatory, antidiabetic, and anti-adipogenic properties have been shown [12,13]. Biological availability of dietary polyphenols is low as compared with micro- and macronutrients. Their absorption in the small intestine amounts only about 5–10%. However, recent studies showed that these phytochemicals exhibit prebiotic properties and antimicrobial activity against pathogenic intestinal microflora [13].

**Figure 1:**
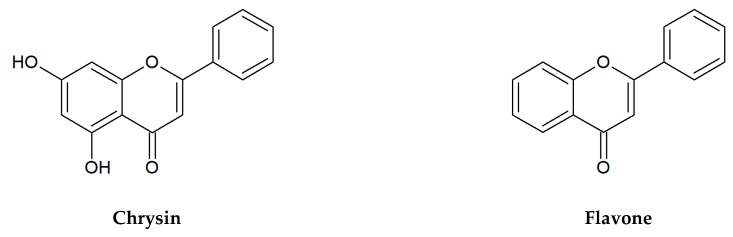

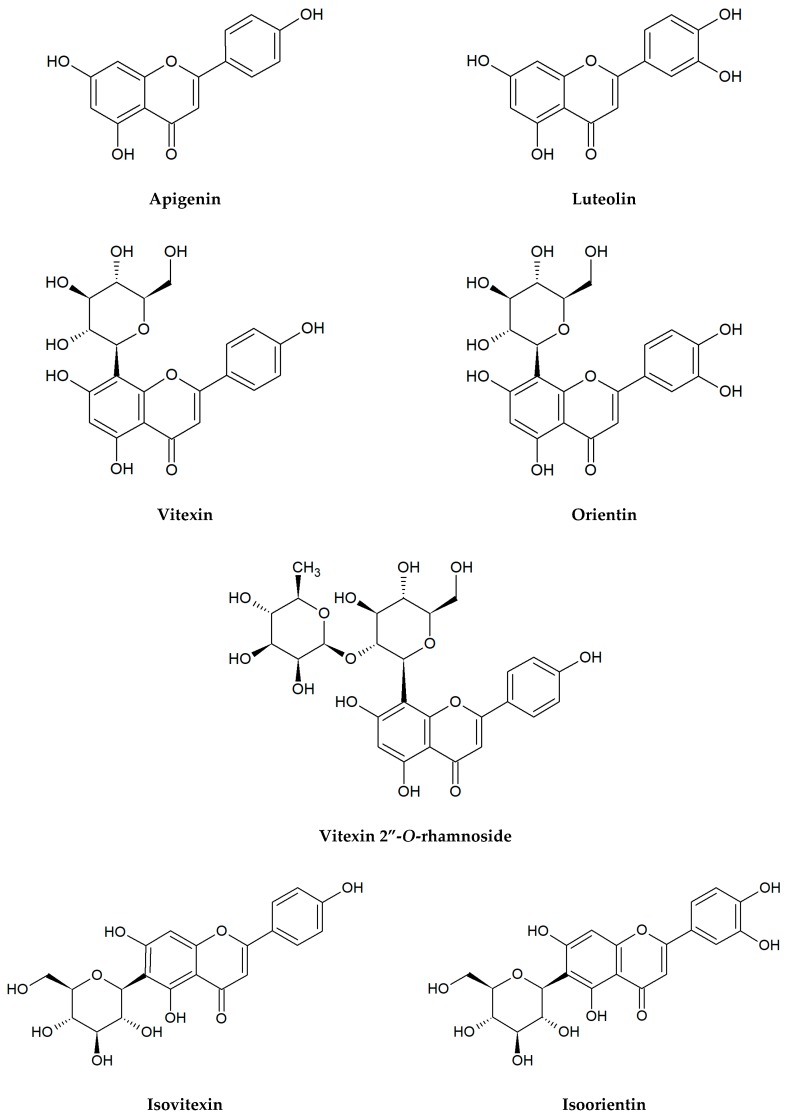

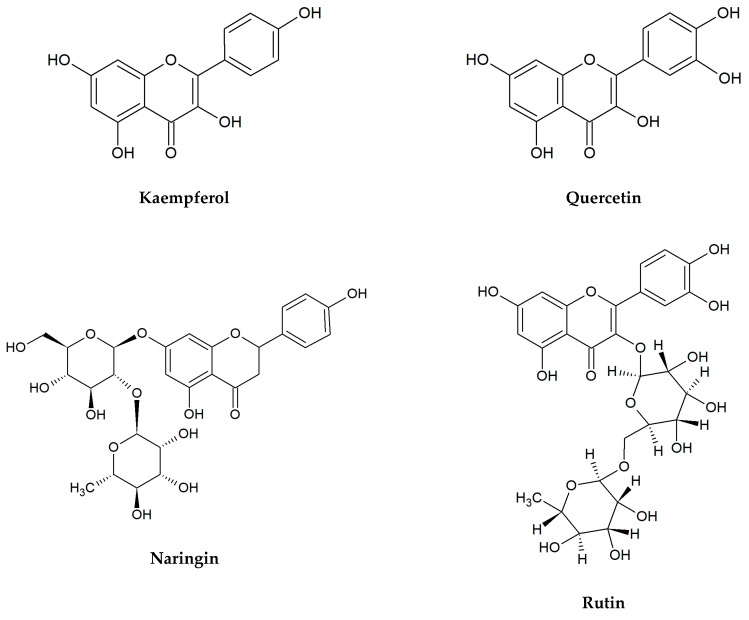
Chemical structures of flavonoids tested in the present research.

Flavonoids selected for our microbiological tests are presented in Figure 1. In the large quantities, they occur in stems and leaves, flowers as well as fruits of the species from the families of Apiaceae, Asteraceae, Betulaceae, Brassicaceae, Ericaceae, Fabaceae, Hypericaceae, Lamiaceae, Liliaceae, Passifloraceae, Polygonaceae, Primulaceae, Ranunculaceae, Rosaceae, Rubiaceae, Rutaceae, Scrophulariaceae, Tiliaceae, and Violaceae. Two flavonols: quercetin, kaempferol, and flavones: apigenin, luteolin belong to the most ubiquitous plant flavonoids [14]. A glycoside form of quercetin—rutin (sophorin, rutoside) is present in the highest concentrations in buckwheat (*Fagopyrum esculentum* Moench), rue (*Ruta graveolens* L.), flower buds of *Styphnolobium japonicum* (L.) Schott (*Sophora japonica* L.), apricots, peaches, and citrus fruits [15,16]. Apigenin derivatives, such as vitexin, isovitexin, and vitexin 2″-*O*-rhamnoside constitute the main bioactive compounds of leaves and flowers of hawthorn (*Crataegus* spp.) [17]. The 8- and 6-*C*-glucosides of luteolin: orientin and isoorientin are reported from different crop plants, including buckwheat, corn silk (*Zea mays* L.), acai fruits (*Euterpe oleracea* Mart., *E. precatoria* Mart.), and Moso bamboo leaves (*Phyllostachys edulis*/Carrière/J.Houz.) [18,19]. In turn, passion fruits (*Passiflora* spp.), skullcap roots (*Scutellaria* spp.) as well as honey and propolis are the main natural sources of chrysin [20,21,22,23]. Naringin is a flavanone glycoside isolated from grapes and citrus fruits, and it imparts a bitter taste to grapefruit juice [24].

**Figure 2:**
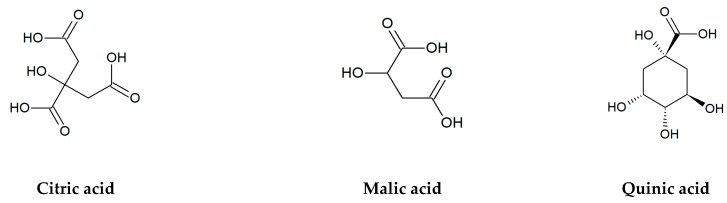

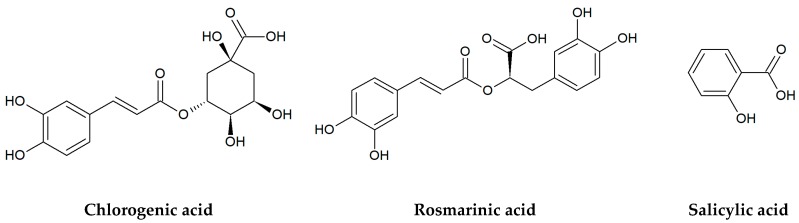
Chemical structures of organic acids tested in the present research.

In addition to flavonoids, a lot of organic acids: both aliphatic and aromatic ones, especially phenolics are the important bioactive compounds of edible and medicinal plants (Figure 2). Among non-aromatic, short-chain hydroxy acids, malic, citric, and quinic acids belong to the most abundant substances with a key role in plant metabolism and physiology. Malic and citric acids are mainly produced in the tricarboxylic acid cycle (Krebs cycle) and, to a lesser degree, in the glyoxylate cycle, while quinic acid is a byproduct of the shikimic acid pathway [4]. High accumulation of these compounds is observed in various berry fruits of wild and cultivated plants from the Ericaceae, Rosaceae, and Grossulariaceae families, including cranberry (*Vaccinium macrocarpon* Aiton and *V. oxycoccos* L.), bilberry (*V. myrtillus* L.), blueberry (*V. corymbosum* L.), blackberry (*Rubus* spp.), raspberry (*Rubus idaeus* L.), black chokeberry (*Aronia melanocarpa*/Michx./Elliott), red currant (*Ribes rubrum* L.), black currant (*Ribes nigrum* L.), and many others [25,26,27,28,29,30]. For example, the total content of citric, malic, and quinic acids in fruits of European cranberry can reach almost 37% of dry matter [31].

Berries are also a rich source of hydroxycinnamic acids and their derivatives, including chlorogenic (5-*O*-caffeoylquinic) and neochlorogenic (3-*O*-caffeoylquinic) acids, which are the esters formed between caffeic (3,4-dihydroxycinnamic) and quinic acids [32,33]. A great amount of chlorogenic acid isomers has been found, among others, in yerba mate (*Ilex paraguariensis* A.St-Hil.), coffee (*Coffea* spp.), and tea plant (*Camellia sinensis*/L./Kuntze) [34]. In turn, rosmarinic acid, an ester of caffeic and 3,4-dihydroxyphenyllactic acids, was isolated for the first time from the rosemary leaves (*Rosmarinus officinalis* L.). It commonly occurs in many aromatic and medicinal plants of the Lamiaceae family, especially mint (*Mentha* spp.) and thyme (*Thymus* spp.) species, lemon balm (*Melissa officinalis* L.), common sage (*Salvia officinalis* L.), oregano (*Origanum vulgare* L.), and sweet basil (*Ocimum basilicum* L.) [35,36,37]. Another well-known secondary metabolite, the phytohormone salicylic acid (SA), is a key signaling compound that participates in the plant response to pathogens, herbivores, and abiotic stress [38]. Natural salicylates such as salicylic acid and salicin (salicyl alcohol glucoside) were found in large amounts in the willow bark (*Salix* spp.), the buds of black poplar (*Populus nigra* L.), elm leaves (*Ulmus* spp.), and meadowsweet herb (*Filipendula ulmaria*/L./Maxim.) [39,40].

Our studies were focused on the estimation of antibacterial activity of selected flavonoids and organic acids widespread in fruits, vegetables, spices, and popular medicinal plants which are very often used for the prevention and treatment of various diseases. For example, many herbal preparations utilized as natural diuretics, and plant extracts with other main pharmacological activities (i.e., drugs against cardiovascular diseases, sedatives, anti-inflammatory agents) exhibit additional beneficial effects by the antimicrobial action [41,42]. Recent data show that flavonoids have protective potential against cutaneous inflammatory reactions and affect wound healing [43,44]. In addition, organic acids (especially citric acid) seem to be of significant importance in the antimicrobial activity and health of the skin [45,46]. In the present studies, we tested the biological activity of chosen flavonoids and organic acids against four widespread pathogens: *Staphylococcus aureus*, *Enterococcus faecalis*, *Pseudomonas aeruginosa*, and *Escherichia coli*. These Gram-positive and Gram-negative bacteria can cause many diseases in humans, including opportunistic infections and belong to the most common etiological factors of the skin and wound infections [47,48].

Microbiological screening tests included 19 plant metabolites from the various flavonoid classes: flavones, flavonols, flavanones, and simple organic acids: aliphatic and aromatic ones. The chosen flavonoids differed in the number of hydroxyl groups on the aromatic rings as well as the presence and position of the sugar group, which gave the opportunity to test the effect of these parameters on the biological activity of the natural compounds.

## 2. Materials and Methods

### 2.1. Chemicals

Chemicals used in this study were purchased from Merck (Sigma-Aldrich, Supelco, Poland). Plant compounds selected for the microbiological tests are presented in Table 1. All substances were dissolved in 20% water solution of dimethyl sulfoxide DMSO (Sigma-Aldrich, Poland) in a final concentration of 1 mg/mL. Additionally, DMSO was used as a negative control, while two antibiotics, ciprofloxacin (Sigma, cat. no. 17850) and gentamicin sulfate (Sigma-Aldrich, cat. no. G1914) as positives.

### 2.2. Bacterial Strains and Antimicrobial Activity

In the in vitro tests, there were investigated clinical isolates of two Gram-positive (*Staphylococcus aureus*, *Enterococcus faecalis*) and Gram-negative bacteria (*Escherichia coli*, *Pseudomonas aeruginosa*). For each species, four strains obtained from the collection of the Department of Medical Microbiology at Poznań University of Medical Sciences (Poland) were tested. None of them were multidrug-resistant. The species of bacteria were grown at 35 °C for 24 h, in tryptone soy agar (TSA; Graso, Poland).

**Figure 3:**
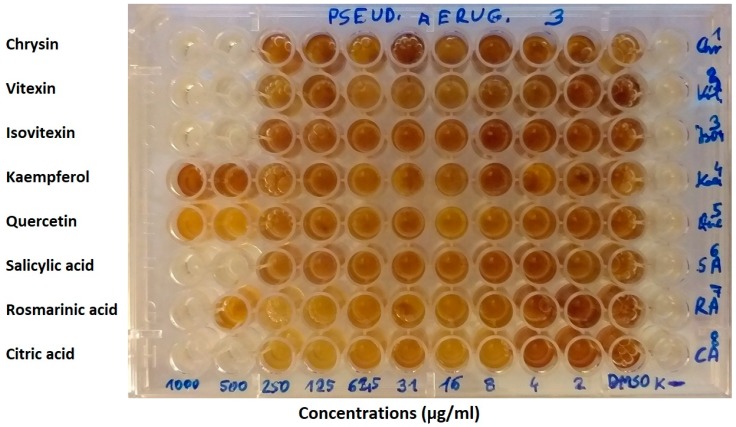
The minimal inhibitory concentrations (MICs) of selected plant substances against *Pseudomonas aeruginosa* strain according to the micro-dilution method.

The minimal inhibitory concentrations (MICs) of selected plant substances were determined by the micro-dilution method using the 96-well plates (Nest Scientific Biotechnology). Studies were conducted according to the Clinical and Laboratory Standards Institute (CLSI) [49], European Committee on Antimicrobial Susceptibility Testing (EUCAST) recommendations [50], and as described in our previous publications [48,51]. Primarily, 90 µL of Mueller–Hinton broth (Graso, Poland) was placed in each well. Serial dilutions of each of the substances were performed so that concentrations in the range of 15.6–1000 µg/mL were obtained. In the initial tests of antibacterial activity of phytochemicals, the lowest concentration amounted to 1.95 µg/mL (Figure 3), while for positive controls (antibiotics) it was 0.98 µg/mL. The inoculums were adjusted to contain approximately 10^8^ CFU/mL bacteria. 10 µL of the proper inoculums were added to the wells, obtaining concentration 10^5^ CFU/mL. The plates were incubated at 35 °C for 24 h, then 20 μL of 1% MTT water solution (3-(4,5-Dimethyl-2-thiazolyl)-2,5-diphenyl-2H-tetrazolium bromide, Sigma-Aldrich) was added to the wells. Next, the plates were incubated 2–4 h at 37 °C. This assay is based on the reduction of yellow tetrazolium salt (MTT) to a soluble purple formazan product [48]. The MIC value was taken as the lowest concentration of the substance that inhibited any visible bacterial growth. The analyses were repeated three times.

In our investigations, we adopted the range of tested concentrations of phytochemicals for the MICs between 15.6 and 1000 µg/mL, although some authors determine the antimicrobial activity of natural compounds at the level of 2000–4000 µg/mL or more [52,53,54]. However, in our opinion, such high values indicate a very weak effect of these substances. During the description of the results, it was taken that the MIC = 250 μg/mL shows a relatively high antibacterial activity of plant chemicals, while the MICs = 500 and 1000 μg/mL mean moderate and low effects, respectively.

## 3. Results

Our research exhibited antibacterial properties of all tested flavonoids and organic acids, but their activity was quite diverse. These compounds were generally more active against Gram-negative than Gram-positive bacteria. The following tendency of microbial sensitivity to plant substances was observed: *E. coli* > *P. aeruginosa* > *E. faecalis* > *S. aureus* (Table 2). Salicylic acid showed the highest biological effect on all bacterial species (MIC = 250–500 μg/mL). However, other chemicals demonstrated a similar activity, especially against *E. coli* and *P. aeruginosa* (MIC = 500 μg/mL). Among 19 investigated phytochemicals, only three: kaempferol, quercetin, and chlorogenic acid had no significant influence on *P. aeruginosa*, while up to 10 compounds were relatively inactive against *S. aureus* (MIC > 1000 µg/mL). It was interesting that the individual strains of a given bacterial species most often did not show differences in the sensitivity to one plant substance. Only salicylic acid, rosmarinic acid, and apigenin exhibited differentiating effects on individual strains.

Although flavonol aglycones kaempferol and quercetin displayed a moderate activity only against *E. coli*, quercetin glycoside rutin demonstrated influence on all strains tested (MIC = 500–1000 μg/mL). A similar activity level was found for the glycosides from the other classes of flavonoids: flavanones (naringin) and flavones (vitexin, isovitexin, vitexin 2″-*O*-rhamnoside, orientin, isoorientin). Differences were determined only in the case of *S. aureus*. Naringin, vitexin and its derivatives showed no significant activity, while orientin and isoorientin were clearly stronger antibacterial agents than rutin.

Among organic acids, the highest variability in the microbiological effect was found against *S. aureus* and *P. aeruginosa*. Some metabolites such as citric, quinic, and rosmarinic acids for *S. aureus*, and also chlorogenic acid for *P. aeruginosa* were relatively inactive. The aliphatic acids: citric, malic and quinic ones showed the same level of activity within individual species of *E. faecalis*, *E. coli*, and *P. aeruginosa* (MIC = 500–1000 μg/mL). In turn, phenolic compounds: chlorogenic, rosmarinic, and salicylic acids exhibited variation within all bacterial species with the MIC values from 250 to above 1000 μg/mL.

## 4. Discussion

In recent years, a rapid increase in the number of studies concerning the antibacterial properties of plant extracts rich in phenolic compounds, including flavonoids and phenolic acids has been observed. However, due to the enormous wealth of species and natural substances, the degree of their examination is very diverse and still insufficient. Particularly, works on the antibacterial activity of individual pure compounds are relatively few. There is a small number of microbiological investigations describing the effects of some common flavonoid glycosides such as vitexin [55,56,57,58], isovitexin [59,60], vitexin 2″-*O*-rhamnoside [61], orientin [62,63], and isoorientin [56,60,62].

In addition, literature data are difficult to compare due to the use of various methods for assessing antibacterial activity, different solvents, and the origin and purity of test compounds, often isolated from various plant extracts [55,57,59,60,62,63,64,65]. Antimicrobial properties of natural chemicals were described not only by the minimum inhibitory concentration (MIC) [33,54,57,62,64,66,67,68,69,70,71,72] and by the minimum bactericidal concentration (MBC) [72], but also by the agar well or disc-diffusion methods [59,60,63,65]. Some authors expressed results as the IC_50_ or MIC_80_ values [33,67,73]. Moreover, the plant substances were tested in various concentrations. The kind of solvent used for the dissolution of pure compounds is the next important point in the assessment of in vitro activity. Although most authors utilized dimethyl sulfoxide, sometimes they did not give its concentration [59,62,63,65] or it is 100% DMSO [54], which may affect the level of antimicrobial activity of the tested solutions. The other solvents used were, for example, acetone [67], chloroform [59], Mueller Hinton II broth [68], and water [71]. In several cases, there was no information about dissolving procedures [57,58,60]. In this context, there is still a need for extensive screening studies that would compare the activity of a large number of plant metabolites against the same bacterial strains by a standardized method.

Our investigations exhibited moderate antibacterial properties of tested flavonoids and organic acids against clinical strains of Gram-negative pathogens: *E. coli* and *P. aeruginosa* (MIC = 500 µg/mL). Among 19 selected plant substances, only three: kaempferol, quercetin, and chlorogenic acid were inactive against *P. aeruginosa* at all concentrations tested (15.6–1000 µg/mL). However, for up to 10 compounds, no significant activity was found against the Gram-positive bacteria *S. aureus*. Additionally, another microorganism from this group *E. faecalis* showed low sensitivity (MIC = 1000 µg/mL) to most analyzed metabolites (Table 2). The above-described observations confirm the results of works which indicate a higher activity of natural plant substances, including flavonoids, against some Gram-negative bacteria than Gram-positive ones, although it is usually considered that this regularity is the opposite [48,55]. The general tendency of bacterial sensitivity to selected plant substances was observed as follows: *E. coli* > *P. aeruginosa* > *E. faecalis* > *S. aureus* (Table 2). Some screening studies showed the greater activity of alkaloids, flavonoids, and phenolic acids especially against *P. aeruginosa*, and also *E. coli* than *S. aureus* [5,55]. However, this relationship seems to have significant limitations and requires further detailed research. For example, all strains of *S. aureus*, *E. coli*, and *P. aeruginosa* tested by us had the same level of sensitivity to flavones chrysin, luteolin, orientin, isoorientin, and some clinical isolates of them to apigenin and salicylic acid. No differences in the inhibitory potency of bacterial growth of above-mentioned species were previously reported, among others, for luteolin, orientin, isoorientin [62], and in the case of *S. aureus* and *E. coli* for chrysin [67], luteolin [74], and glycosides of quercetin hyperoside and rutin [72].

Numerous studies allow to state that in antibacterial mechanisms of flavonoids are included mainly: inhibition of synthesis of nucleic acid, inhibition of cytoplasmic membrane function by influence the biofilm formation, porins, permeability, and by interaction with some crucial enzymes [6,8,75,76]. It was shown that apigenin inhibits the DNA gyrase of *E. coli* [77], and has inhibitory effects on the formation of *E. coli* biofilm [78]. Recently, a liposomal formulation of apigenin was examined, and it was observed increasing of its antibacterial property by the interaction of apigenin liposomes with the membrane of tested bacteria resulted in the lysis of the bacterial cells. Comparison of results exhibited much greater efficiency of liposomal apigenin against both Gram-positive and Gram-negative bacteria: *B. subtilis* (MIC = 4 µg/mL), *S. aureus* (MIC = 8 µg/mL), and *E. coli* (MIC = 16 µg/mL), *P. aeruginosa* (MIC = 64 µg/mL) [68]. Other flavones, including apigenin *C*-glucosides such as vitexin and isovitexin, have also been tested in order to study their effect on bacterial surface hydrophobicity and biofilm formation [57,58,59]. Das et al. [58] reported that vitexin reduces the hydrophobicity of cell surface and membrane permeability of *S. aureus* at the sub-MIC dose of 126 µg/mL. This flavone down-regulated the *ica*AB and *agr*AC gene expression showing antibiofilm activity and bactericidal effect. In similar work, Das et al. [57] demonstrated that vitexin exerts the MIC of 260 µg/mL against *P. aeruginosa*, and exhibits moderate antibiofilm activity. In turn, isovitexin (200–500 µg/mL) decreased the adhesion of methicillin-sensitive *S. aureus* ATCC 29213, and simultaneously increased the adhesion of two strains of *E. coli* [59]. Currently, it was shown that isovitexin has the potent antibacterial properties described as the diameter of the zone of growth inhibition (ZOI) for *B. subtilis* (19.5 mm), *P. aeruginosa* (17.5 mm), *E. coli* (14.1 mm), and *Staphylococcus aureus* (12.8 mm). The even stronger activity was found for isoorientin (luteolin *C*-glucoside), and it was as follows: *B. subtilis* (20.1 mm), *P. aeruginosa* (19.1 mm), *S. aureus* (18.7 mm), and *E. coli* (14.8 mm) [60].

Microbiological literature provides interesting data on the mechanism of action of two main flavonols: kaempferol and quercetin. It was shown that quercetin increases the cytoplasmic membrane permeability of *S. pyogenes* which resulted in the inhibitory influence on this Gram-positive bacterium at the MIC value of 128 µg/mL [79]. Moreover, in this study, the synergistic effect of quercetin with antibiotic ceftazidime was observed. Barbieri et al. [6] concluded that this flavonol is active not only against Gram-positive pathogens: *S. aureus*, *S. haemolyticus*, and *S. pyogenes*, but also against Gram-negative ones: *E. coli* and *K. pneumoniae*. Additionally, Betts et al. [80] showed a strongly inhibiting effect against methicillin-resistant *S. aureus*, which was significantly increased in the presence of epigallocatechin gallate. Studies of the mechanism of antimicrobial action allowed to state that quercetin diacyl glycosides show dual inhibition of DNA gyrase and topoisomerase IV [81]. In turn, our investigations exhibited the moderate effect of these plant metabolites against *E. coli* (MIC = 500 µg/mL), and lack of significant activity in the case of *S. aureus* (MIC > 1000 µg/mL). Research of Chen and Huang [82] concerning quercetin and kaempferol reported inhibition of the interaction of DNA B helicase of *K. pneumoniae* with deoxynucleotide triphosphates (dNTPs). Further study showed that the ATPase activity of this helicase *Kp*DnaB was decreased to 75% and 65% in the presence of quercetin and kaempferol, respectively [83]. In the next work, Huang et al. [84] observed that kaempferol inhibits the DNA PriA helicase of *S. aureus*, and these results showed that the concentration of phosphate from ATP hydrolysis by this DNA helicase was decreased to 37% in the presence of 35 µM kaempferol. Thus, it was summarized that kaempferol can bind to DNA helicase and then inhibit its ATPase activity and this is a new mechanism of action for this chemical compound. According to the results, this flavonol may be taken into consideration as an active natural molecule in the development of new antibiotics against *S. aureus* [84]. Currently, Huang [73] demonstrated the inhibitory effect of kaempferol on the activity of a dihydropyrimidinase from *P. aeruginosa* with the IC_50_ value of 50 ± 2 μM.

Nowadays, it is believed that the structure-activity relationship in the antimicrobial effect of flavonoids should be further examined because it is a very large group of compounds, and many issues have not yet been clarified. Xie et al. [75] concluded that hydroxyl groups at special positions on the aromatic rings of flavonoids improve the antibacterial effect. Flavonoids have the C_6_-C_3_-C_6_ carbon structure consisting of two phenyl rings (A and B) and a heterocyclic ring (C). Generally, it was observed that at least one hydroxyl group in the ring A (especially at C-7) is vital for the antibacterial activity of flavones, and in another position such as C-5 and C-6 can increase this biological effect [85]. In this context, it is interesting to compare our results regarding the antibacterial activity of flavones with the hydroxyl groups at C-5 and C-7 (chrysin, apigenin, luteolin, and their glycosides) and flavone devoid of them. Just like other chemicals from this flavonoid class, flavone showed moderate inhibitory influence on the growth of *E. coli* and *P. aeruginosa* (MIC = 500 µg/mL). The same level of flavone activity was found against *E. faecalis*, and it was the highest value among the flavonoids tested. Only against *S. aureus*, the above-mentioned substance was inactive at concentrations tested (15.6–1000 µg/mL). Furthermore, we observed that a number of hydroxyl groups at two aromatic rings do not correspond with higher antimicrobial activity of flavonoids, i.e., quercetin has five hydroxyl groups, but it was not active against *E. faecalis*, *S. aureus*, and *P. aeruginosa*. In addition, some studies displayed a low effect of quercetin on *B. subtilis*, *E. cloacae*, *E. coli*, and *K. pneumoniae* [86]. The structure-activity relationships of flavonoids were discussed by Xie et al. [87], and it was summarized that two hydroxyl substituents on C-5 and C-7 of ring A of quercetin, rutin, and naringenin lead to their antibacterial activities. Moreover, it was found that the saturation of the C_2_=C_3_ double bond (in naringin) increased the antibacterial activity. However, in our study naringin was the most active against *P. aeruginosa* only in comparison with kaempferol and quercetin. On the other side, a recent study showed that the presence of glycosyl conjugated groups to polyphenols may reduce antibacterial activity [88]. We showed that glycosides of flavonoids (vitexin, vitexin 2″-*O*-rhamnoside, isovitexin, orientin, isoorientin, naringin, rutin) have some antibacterial effects (Table 2). The aglycone apigenin exhibited higher activity against *S. aureus* in comparison with its glycosides vitexin, isovitexin, and vitexin 2″-*O*-rhamnoside, however the aglycon luteolin had the same antibacterial effects on all bacterial strains as its *C*-glucosides orientin and isoorientin.

According to the literature, the level of sensitivity of the bacterial species studied by us to plant substances is very diverse and strongly depends not only on the type of active compound but also on the selected strains, as shown by comparative analyses in this regard [5,53]. It may also affect large discrepancies in the results between individual investigations. Some literature data suggest that standard strains are generally much more sensitive to antibiotics and natural plant compounds than current clinical isolates. For example, the MIC values of quercetin, apigenin, naringin, chlorogenic, and quinic acids for *E. coli* ATCC 35218, *P. aeruginosa* ATCC 10145, *S. aureus* ATCC 25923, and *E. faecalis* ATCC 29212 reached 2–16 µg/mL, while for the clinical strains it ranged between 32 and 128 µg/mL or above this [5]. In turn, research conducted by Su et al. [53] showed a slightly higher sensitivity of some clinical isolates of methicillin-resistant *S. aureus* to luteolin and quercetin (MIC = 31.2–62.5 µg/mL) than methicillin-sensitive strains (MIC = 125 µg/mL). In the study of Morimoto et al. [89], quinolone-resistant *S. aureus* Mu50 was much more sensitive to apigenin (MIC = 4 µg/mL) than quinolone-susceptible *S. aureus* strain FDA 209P (MIC > 128 µg/mL). Compared to the above-cited works [5,89], it was interesting that apigenin and chlorogenic acid were practically inactive (MIC > 4000 µg/mL) against all 34 strains of *S. aureus* tested by Su et al. [53]. Our investigations exhibited the moderate or weak activity of these two compounds against *S. aureus* (MIC = 500–1000 µg/mL). A recent review of the literature [33] showed that chlorogenic acid has a broad spectrum of antimicrobial activity, but its effect is very diverse. This phenolic acid strongly inhibited the growth of *E. faecalis* (MIC = 64 µg/mL), while it was inactive against *P. aeruginosa* (MIC_80_ = 10,000 µg/mL). For *S. aureus* and *E. coli*, its MIC values ranged from 40–80 to 10,000 µg/mL. The above data are largely consistent with the results of the current work (Table 2). We exhibited the moderate activity of chlorogenic acid against *E. coli* (MIC = 500 µg/mL) and confirmed the lack of significant influence of this substance on *P. aeruginosa* at the concentrations tested (MIC > 1000 µg/mL).

In addition to chlorogenic acid, we also studied the biological influence of other phenolic acids: rosmarinic and salicylic ones. In addition, their antibacterial activity was compared with some aliphatic acids: citric, malic, and quinic. Generally, there were no clear differences in the activity of these two groups of substances. However, the simple phenolic compound salicylic acid showed the highest activity with the MIC values of 250–500 µg/mL. Many studies proved that rosmarinic acid has an antimicrobial effect on Gram-positive and Gram-negative bacteria [65,71,90]. Sometimes, the level of this activity was not high. Recently, Akhtar et al. [65] indicated the moderate growth inhibition zones of clinical isolates of *P. aeruginosa* (13 mm in diameter), *S. aureus* (12 mm), *Proteus vulgaris* (11 mm), and *E. coli* (10 mm) at 1 µg/mL concentration of rosmarinic acid. Similarly, Matejczyk et al. [71] observed a not very strong antibacterial effect of this phenolic acid on *E. coli* (MIC > 250 µg/mL), *Bacillus* sp. (MIC > 500 µg/mL), *S. epidermidis* (MIC > 500 µg/mL), and *S. pyogenes* (MIC > 500 µg/mL) in comparison with an antibiotic kanamycin (MIC > 100 µg/mL). In turn, Ekambaram et al. [69] demonstrated the MIC values of rosmarinic acid against *S. aureus* and MRSA on the level of 800 and 10,000 µg/mL, respectively. Blaskovich et al. [54] carried out experimental research and made a critical review of the antimicrobial activity of salicylic acid. Results of these studies demonstrated that salicylic acid was practically inactive against various bacterial strains, including *B. subtilis* ATCC 6633, *E. faecalis* ATCC 29212, *S. aureus*, MSSA ATCC 25923, and *S. pneumoniae* ATCC 33400 (MIC = 32,000 µg/mL). The antibacterial properties are relatively well known for small aliphatic molecules tested by us: citric, malic, and quinic acids [91,92,93]. Investigations concerning the effect of quinic acid on cellular functions of *S. aureus* demonstrated that this organic acid could significantly decrease the intracellular pH and ATP concentration, and also reduce the DNA content [93]. Citric acid was previously shown to have the MICs of 900 µg/mL for *S. aureus* and 1500 µg/mL for *E. coli*, and to be very effective in the treatment of chronic wound infections in a dose of 3 g of citric acid dissolved in 100 mL of distilled water [91]. In turn, Gao et al. [85] demonstrated no clear activity of citric and malic acids against *E. coli* (MIC = 1667 and 2000 µg/mL, respectively) *B. subtilis* (MIC = 2000 µg/mL), and *S. suis* (MIC = 8000 and 6667 µg/mL). However, Jensen et al. [92] exhibited that cranberry juice and its main compounds (citric, malic, quinic, and shikimic acids) reduce *E. coli* colonization of the bladder. These organic acids decreased bacterial levels when they were administered together or in a combination of malic acid and citric or quinic ones. Our research confirmed the antibacterial activity of citric, malic, and quinic acids not only against *E. coli* (MIC = 500 µg/mL), but also against *P. aeruginosa* and *E. faecalis* (Table 2).

## 5. Conclusions

Our research confirmed the antibacterial activity of all tested plant compounds. With the exception of kaempferol and quercetin, they showed a biological effect against clinical strains of 3–4 bacterial species. Microbiological screening of flavonoids and organic acids allowed to exhibit some interesting details and relationships. First of all, these metabolites were generally more potent against Gram-negative bacteria: *E. coli* and *P. aeruginosa* than Gram-positive ones: *E. faecalis* and *S. aureus*. On the other hand, the comparative study of antibacterial activity of flavone, chrysin, apigenin, and luteolin demonstrated that the presence of hydroxyl groups in the phenyl rings A (C-5, C-7) and B (C-3′, C-4′) usually did not affect the activity level of flavones. Only in the case of *S. aureus*, a clear increase in the activity of the hydroxy derivatives of flavone was observed. Similarly, the presence and position of the sugar group in the flavone glycosides generally had no effect on the MIC values.

A comparison of our results with the literature data exhibited that the level of sensitivity of the bacterial species to plant substances is very diverse, and strongly depends not only on the type of active compounds but also on the strains tested. Moreover, it seems that current clinical isolates are generally much less sensitive to the natural plant metabolites than standard strains. Numerous standard strains have been isolated many years ago, therefore, with the currently growing resistance of bacteria, their use for the screening microbiological tests is limited. In our investigations, we found the moderate or even low activity of flavonoids and organic acids compared to the traditional antibiotics and some plant substances. However, examples of the use of natural compounds with a relatively low in vitro activity in the treatment of urinary tract infections, chronic wound infections, etc. or as food additives show that widely distributed flavonoids and organic acids could find broad practical applications.

## Figures and Tables

**Table 1:** Plant pure substances used in the microbiological assays.

No	Merck (Sigma-Aldrich, Supelco)	CAS No	PubChem CID	Purity
1	Apigenin	520-36-5	5280443	≥95.0% (HPLC)
2	Chrysin	480-40-0	5281607	≥98.0% (HPLC)
3	Flavone	525-82-6	10680	≥99.0%
4	Isoorientin	4261-42-1	114776	≥98.0% (HPLC)
5	Isovitexin	38953-85-4	162350	≥98.0% (HPLC)
6	Kaempferol	520-18-3	5280863	≥97.0% (HPLC)
7	Luteolin	491-70-3	5280445	≥97.0% (HPLC)
8	Naringin	10236-47-2	442428	≥95.0% (HPLC)
9	Orientin	28608-75-5	5281675	≥98.0% (HPLC)
10	Quercetin	117-39-5	5280343	≥95.0% (HPLC)
11	Rutin	153-18-4	5280805	≥95.0% (HPLC)
12	Vitexin	3681-93-4	5280441	≥95.0% (HPLC)
13	Vitexin 2″-*O*-rhamnoside	64820-99-1	5282151	≥98.0% (HPLC)
14	Chlorogenic acid	327-97-9	1794427	≥95.0% (HPLC)
15	Citric acid	77-92-9	311	≤100%
16	Malic acid	6915-15-7	525	≤100%
17	Quinic acid	77-95-2	6508	analytical standard
18	Rosmarinic acid	20283-92-5	5281792	≥98.0% (HPLC)
19	Salicylic acid	69-72-7	338	≥99.0%

**Table 2:** Antibacterial activity of selected plant substances against Gram (+) and Gram (−) bacteria.

Plant Substance	Tested Bacteria			
	*Staphylococcus aureus*	*Enterococcus faecalis*	*Escherichia coli*	*Pseudomonas aeruginosa*
	MIC (µg/mL)			
Kaempferol	>1000	>1000	500	>1000
Quercetin	>1000	>1000	500	>1000
Rutin	1000	1000	500	500
Naringin	>1000	1000	500	500
Flavone	>1000	500	500	500
Chrysin	500	1000	500	500
Apigenin	500, 1000 (3x)	1000	500	500
Vitexin	>1000	1000	500	500
Isovitexin	>1000	1000	500	500
Vitexin 2″-*O*-rhamnoside	>1000	1000	500	500
Luteolin	500	1000	500	500
Orientin	500	1000	500	500
Isoorientin	500	1000	500	500
Citric acid	>1000	1000	500	500
Malic acid	1000	1000	500	500
Quinic acid	>1000	1000	500	500
Chlorogenic acid	1000	1000	500	>1000
Rosmarinic acid	>1000	1000	500	500 (2x), 1000 (2x)
Salicylic acid	250 (2x), 500 (2x)	500	250 (3x), 500	500
Median	>1000	1000	500	500
20% DMSO (negative control)	>1000	>1000	>1000	>1000
Ciprofloxacin (positive)	<1	<1	<1	<1
Gentamicin sulfate (positive)	<1	<1–62.5	<1–3.9	<1
